# Nickel-Doped ZnO Nanowalls with Enhanced Electron Transport Ability for Electrochemical Water Splitting

**DOI:** 10.3390/nano11081980

**Published:** 2021-07-31

**Authors:** Bing-Chang Jiang, Sheng-Hsiung Yang

**Affiliations:** Institute of Lighting and Energy Photonics, College of Photonics, National Yang Ming Chiao Tung University, Tainan 71150, Taiwan; ben0658228@gmail.com

**Keywords:** nickel-doped, zinc oxide nanowalls, surface roughness, pore size, electrochemical water splitting

## Abstract

This article reports on the growth of 3 mol% nickel (Ni)-doped zinc oxide nanowalls (ZnO NWLs) using the hydrothermal method. Morphological investigation as well as electrical conductivity of the undoped and Ni-doped ZnO NWLs was also discussed. The surface roughness of the formed ZnO NWLs was reduced after Ni-doping. The pore size of Ni-doped ZnO NWLs can be controlled by changing the concentration of hexamethylenetetramine (HMT). As the HMT concentration increased, the pores became larger with increasing surface roughness. The electrical conductivity of the electron-only device based on the Ni-doped ZnO NWLs was higher than that of the undoped one, and it was decreased with increasing the HMT concentration. Our results reveal that Ni-doping and adjustment of the HMT concentration are two key approaches to tune the morphology and electrical properties of ZnO NWLs. Finally, the undoped and Ni-doped ZnO NWLs were used as the catalyst for electrochemical water splitting. The Ni-doped ZnO NWLs with the HMT concentration of 1 mM showed the highest electrochemical performance, which can be attributed to the increased surface area and electrical conductivity.

## 1. Introduction

The continuous development in the growth of nanostructured metal oxides is a key issue for optoelectronic applications in sensors [1], transistors [2], and others [3,4]. A deeper understanding of growth mechanism of metal oxide nanostructures with high quality and low cost is extremely important for future production. At present, common metal oxides include titanium oxide (TiO_2_), zinc oxide (ZnO), and tungsten oxide (WO*_x_*). The electron mobility of TiO_2_ and WO*_x_* is reported to be 0.1–4 and 12 cm^2^/Vs [5,6], respectively, while ZnO possesses 10-fold higher electron mobility (110–138 cm^2^/Vs) with lower calcination temperatures [5,7]. It is reported that ZnO can be obtained from its precursor film by annealing at 150–200 °C [5]; however, this requires relatively higher calcination temperatures of up to 500 °C to obtain high-quality WO*_x_* [6]. Specific nanostructures of the above three materials can be grown via the hydrothermal method, and the growth temperature of ZnO is the lowest among the three oxides. For example, TiO_2_ or WO*_x_* nanorods can be grown on the substrate from their precursor solutions at 170 °C with the aid of Teflon-lined autoclaves [8,9], while ZnO nanorods are easier to grow on the substrate at a lower temperature of 95 °C [10]. Besides, ZnO possesses many fascinating properties, including earth abundance, non-toxicity, high transmittance in the visible range, and large piezoelectric response [11,12,13]. Highly ordered ZnO nanostructures such as nanorods [14], nanowires [15,16,17], nanoflowers [18], and nanowalls (NWLs) [19] have been prepared by chemical vapor deposition, metal organic chemical vapor deposition, the molecular beam epitaxy method, and microwave synthesis [20]. Those processes require expensive equipment, high reaction temperatures, and complicated parameter settings that usually lead to high production costs. In order to prevent those drawbacks, the low-cost hydrothermal method has been gradually adapted, which permits facile fabrication and the use of flexible substrates. To date, numerous studies concerning ZnO nanostructures using the hydrothermal method have been published in the literature in relation to nanorods [21,22,23], nanowires [24,25,26], nanoflowers [27], and NWLs [28,29,30]. ZnO nanowires and nanoflowers are usually inhomogeneously distributed on the substrate with random layer thickness, with difficulties in device fabrication. ZnO nanorods and NWLs provide two-dimensional structures and both can be grown vertically on the substrate. NWLs have a much thinner wall thickness and a larger surface area [30], which is beneficial for carrier extraction and transport to reach high device efficiency. As a result, this study is focused on preparation as well as the morphological and electrical investigation of ZnO NWLs.

ZnO NWLs are reported to have intertwined honeycomb or sponge-like nanostructures [5,30]. There are two ways to grow NWLs on the substrate via the hydrothermal method as per the previous reports [28,29]. The first way contains three steps, including the deposition of ZnO seed layer, growth of ZnO nanorods, and etching of nanorods with an aqueous KOH solution to form NWLs. The formation of NWLs is ascribed to defect-selective dissolution in (001) planes and etching along the c-axis by OH^−^ in chemical bath. Shin et al. firstly prepared ZnO NWLs by etching in KOH solution [31]. A ZnO sidewall of approximately 30 nm was formed by the partial dissolution of the (001) surface of the ZnO thin film after etching for 15 min. The formed ZnO NWLs were utilized as the electron transport layer (ETL) for fabricating inverted polymer solar cells, revealing a power conversion efficiency (PCE) value of 1.8%. Liang et al. also prepared ZnO NWLs using the similar etching technique to form much denser structures [28]. The inverted polymer solar cells based on dense ZnO NWLs reached a PCE value of 2.14%. The second way also involves three steps, including the deposition of ZnO compact layer, thermal evaporation of an aluminum (Al) thin layer, and growth of ZnO NWLs in chemical bath. The formation mechanism of NWLs is attributed to prohibition of ZnO growth along the [001] direction while allowing the occurrence of lateral growth, as proposed by Chu and coworkers [27]. The ZnO NWLs were grown on silicon wafer using an aluminum mask in conjunction with a hydrothermal method, which produce two cathodoluminescence peaks at 378 and 560 nm. They also found that ZnO nanoflowers would form on NWLs by controlling the concentration of hexamethylenetetramine (HMT) and reaction time. Tang et al. also used the same method to grow ZnO NWLs with a large number of sponge-like pores [5]. The thickness of the film and sidewalls was measured to be 320 and 30–80 nm, respectively. The formed ZnO NWLs were utilized as the ETL for the fabrication of perovskite solar cells with the configuration of indium tin oxide (ITO)/ZnO NWLs/perovskite/Spiro-OMeTAD/Ag. The optimized device achieved a moderate PCE value of 13.6%. Feng et al. changed the molar ratio of the two starting materials zinc nitrate hexahydrate and HMT to prepare ZnO NWLs [10]. When the molar ratio of zinc nitrate hexahydrate and HMT was controlled to 1:1 or 2:1, similar NWL structures were formed. As the concentration of HMT was increased (molar ratio = 1:2), flower-like ZnO nanoparticles on top of NWLs were observed. It is seen that HMT is a popular chemical compound used in the literature to control or modify the structure of ZnO nanomaterials [20]. To date, changing the concentration of HMT has led to complex ZnO nanostructures. The preparation of ZnO NWLs with uniform thickness and controllable pore size is still undergoing.

In the field of water splitting, several state-of-the-art works have been reported. Reddy et al. utilized 1 mol% Ni-doped ZnO structures as the catalyst that showed the maximum photoelectrochemical activity under visible light illumination [32]. The maximum current density of ~3.28 mA/cm^2^ at a 0.1 M NaOH electrolyte demonstrated a superior charge separation. Khan et al. fabricated Ni-doped ZnO thin films for efficient overall water splitting [33]. The incorporation of Ni^2+^ significantly enhanced the photoelectrochemical performance by improving the charge transport properties and conductivity of the ZnO host matrix. In this work, we first demonstrate the preparation of nickel (Ni) doped ZnO NWLs via the hydrothermal method. The increment in electrical conductivity of Ni-doped ZnO NWLs was verified from electrical measurement of electron-only devices. In order to control the pore size of ZnO NWLs, the molar concentration of HMT was controlled from 1 to 5 mM, while the molar concentration of zinc nitrate hexahydrate was fixed to 0.025 M in our system. The effects of HTM concentration on morphology and surface roughness of ZnO NWLs were examined by scanning electron microscopy (SEM) and atomic force microscopy (AFM). We found that the pore size of NWLs could be fine-tuned by the introduced HMT amount. Moreover, the flower-like aggregates on ZnO NWLs were completely suppressed by controlling the HMT concentration at lower than 7 mM. Apart from SEM observations, we provided a series of AFM topographic images as well as surface roughness data to support our results, which have rarely been reported in the literature. The elemental analysis of the undoped and Ni-doped ZnO NWLs was verified by the energy-dispersive spectroscopy (EDS) technique. The transmission and absorption properties of different types of ZnO NWLs were also investigated. Finally, ZnO NWLs were used as the catalyst for water splitting application. A linear sweep voltammetry (LSV) experiment was applied to study electrochemical activity of the prepared ZnO NWLs.

## 2. Materials and Methods

### 2.1. Materials

ITO glass substrates (7 Ω/□) were purchased from Merck (Darmstadt, Germany). The original size of ITO substrates was 37 × 40 cm^2^, and they were cut into small pieces of 2 × 2 cm^2^ for further use. Zinc acetate dihydrate (C_4_H_10_O_6_Zn, purity 98.0–101.0%), zinc nitrate hexahydrate (H_12_N_2_O_12_Zn, purity 99%), and nickel nitrate hexahydrate (H_12_N_2_NiO_12_, purity 98%) were all purchased from Alfa Aesar (Ward Hill, MA, USA). HMT (C_6_H_12_N_4_, purity 99%) was purchased from SHOWA (Osaka, Japan). Monoethanolamine (C_2_H_7_NO, purity 99%) and 2-methoxyethanol (C_3_H_8_O_2_, purity 99+%) were bought from Acros (Geel, Belgium). Other reagents and solvents were bought from Alfa Aesar (Ward Hill, MA, USA) and used without further purification.

### 2.2. Preparation of ZnO NWLs

**Figure 1:**
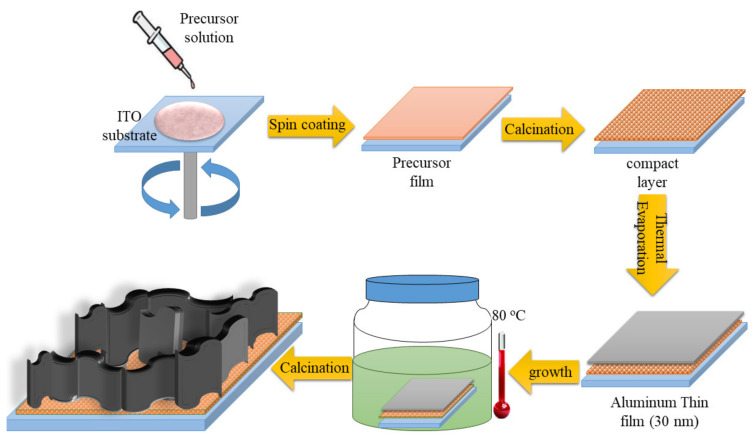
Schematic illustration of the formation of ZnO NWLs.

Prior to the growth of ZnO NWLs, the ITO substrates were cleaned sequentially with deionized (DI) water, acetone and isopropanol for 20 min each in ultrasonic bath, followed by UV-ozone exposure for 30 min. The ultrasonic cleaner (model D150, 150 W) was manufactured by DELTA Ultrasonic Co., Ltd. (New Taipei City, Taiwan). ZnO NWLs were grown using a 3-step deposition method. First, the ZnO compact layers were formed by spin-coating from a precursor solution at 3000 rpm for 30 s on the ITO substrates. The precursor solution was prepared by mixing zinc acetate dihydrate (0.2195 g, 0.1 mol) and monoethanolamine (0.061 g, 0.1 mol) in 10 mL of 2-methoxyethanol and heating at 60 °C for 2 h with stirring. After spin-coating, the precursor films were immediately calcinated at 300 °C for 30 min in air. The high-temperature oven (model OV-455) was manufactrured by PANCHUN Scientific Corp. from Taiwan. Second, a 30 nm-thick aluminum layer was deposited on the ZnO compact layer by thermal evaporation. Third, the NWLs were grown in a growth solution at 80 °C for 50 min. Two separate solutions were prepared in advance. One was zinc nitrate hexahydrate (0.144 g, 0.48 mmol) dissolved in 20 mL of DI water, and the other was HMT dissolved in 20 mL of DI water. The concentration and weight of HMT were controlled to be 1 mM (0.0028 g), 3 mM (0.0084 g) and 5 mM (0.014 g) to study the effect of HMT concentration on the morphology of ZnO NWLs. The zinc nitrate hexahydrate solution was then poured into the HMT solution to form the growth solution. For Ni doping, nickel nitrate hexahydrate (0.0043 g, 0.0147 mmol) was added in the zinc nitrate hexahydrate solution. The Ni-doping content was 3 mol% relative to zinc nitrate hexahydrate. After growth, the substrates were taken out, rinsed with DI water, and purged with nitrogen flow, followed by calcination at 300 °C for 30 min in air to form final ZnO NWLs. The schematic illustration of the ZnO NWLs formation is depicted in Figure 1.

### 2.3. Characterization Methods

The top-view and cross-sectional SEM micrographs of samples were investigated with an ultra-high resolution ZEISS Auriga SEM. The EDS analyzer (Quantax, BRUKER, Billerica, MA, USA) was also equipped on the same SEM for elemental analysis. The surface morphology and roughness of ZnO NWLs were performed using a Bruker Innova AFM (Billerica, MA, USA). The transmission and absorption spectra of samples were recorded with a Princeton Instruments Acton 2150 spectrophotometer equipped with a Xe lamp as the light source. The current–voltage (I–V) characteristics of electron-only devices were measured using an Agilent 4155C semiconductor parameter analyzer. The LSV experiment was performed on an AUTOLAB PGSTAT30 potentiostat for electrochemical water splitting test. The electrochemical cell was composed of 3 electrodes, with Ag/AgCl (Metrohm AG) as the reference electrode, Pt wire as the counter electrode, and ZnO NWLs samples as the work electrode. The electrolyte was 0.1 M KOH aqueous solution. The scan rate and active area of each sample were 5 mV/s and 1 cm^2^, respectively.

## 3. Results and Discussion

### 3.1. Morphological Investigation

**Figure 2:**
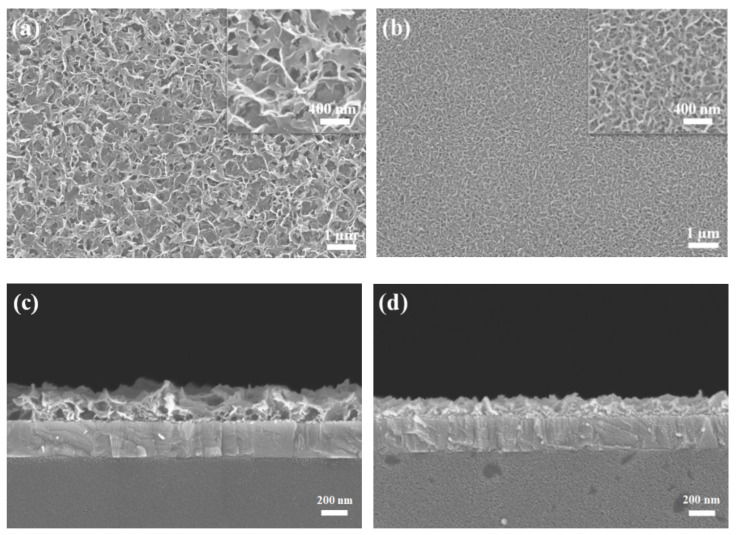
Top-view and cross-sectional SEM images of the (**a**,**c**) undoped and (**b**,**d**) Ni-doped ZnO NWLs.

**Figure 3:**
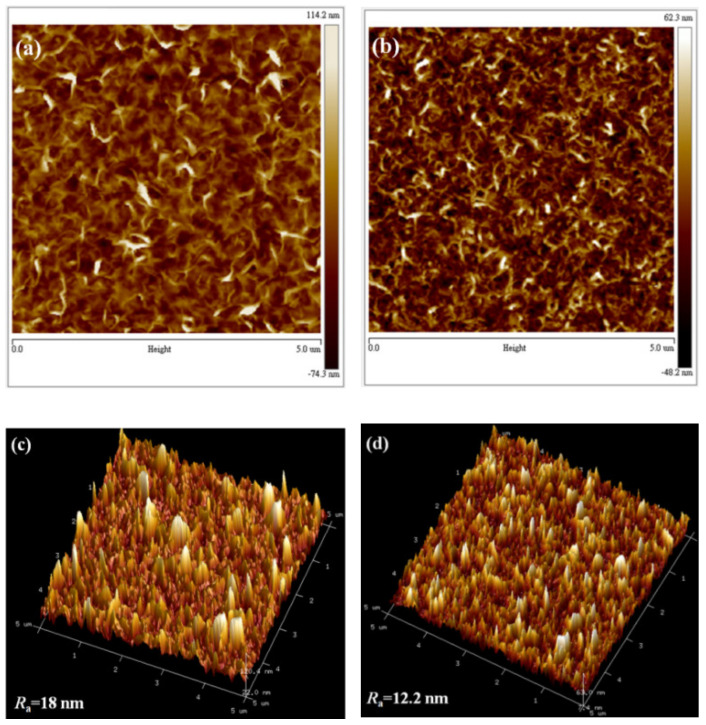
AFM topographic and 3D images of the (**a**,**c**) undoped and (**b**,**d**) Ni-doped ZnO NWLs.

The top-view and cross-sectional SEM micrographs of the undoped and Ni-doped ZnO NWLs with the HMT concentration of 1 mM are shown in Figure 2. The morphology of our undoped ZnO NWLs looks similar with other reports in the literature [11,14,29]. Compared with the undoped ZnO, Ni-doped ZnO NWLs have more staggered networks, indicative of greater surface area. Both the undoped and Ni-doped ZnO NWLs showed sponge-like nanostructures through SEM observation. Moreover, the pores of Ni-doped ZnO NWLs are obviously smaller and uniform than the undoped one, as shown in the insets in Figure 2a,b. The thickness of the undoped and Ni-doped ZnO NWLs is estimated to be 250 and 150 nm, respectively, from the cross-sectional SEM images in Figure 2c,d. Apart from morphology, surface roughness is also an important issue for the construction of optoelectronic devices with high performance. In this part, AFM technique was utilized to investigate topographic images and average roughness (R_a_) of the prepared ZnO NWLs. The corresponding AFM images and R_a_ values are revealed in Figure 3. It is clearly seen that both the undoped and Ni-doped ZnO NWLs have inter-connected wall structures and the latter possesses denser morphologies, which is in good accordance with SEM observations. The R_a_ values of the undoped and Ni-doped ZnO NWLs were estimated to be 18 and 12.2 nm, respectively, revealing that Ni-doping would decrease surface roughness of ZnO NWLs. From literature survey, we learn that AFM topographic images of ZnO NWLs are less reported, while corresponding R_a_ value has not been disclosed so far. It is well known that lower roughness has a positive impact for reducing leakage current and enhancing device performance. This is the first time that complete AFM images and roughness measurements of ZnO NWLs have been demonstrated.

**Figure 4:**
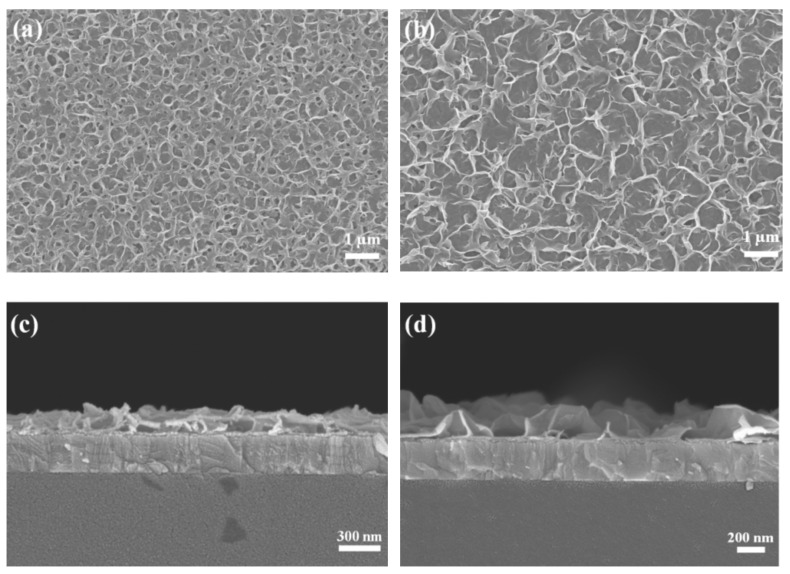
Top-view and cross-sectional SEM images of the Ni-doped ZnO NWLs with HMT concentrations of (**a**,**c**) 3 and (**b**,**d**) 5 mM.

**Figure 5:**
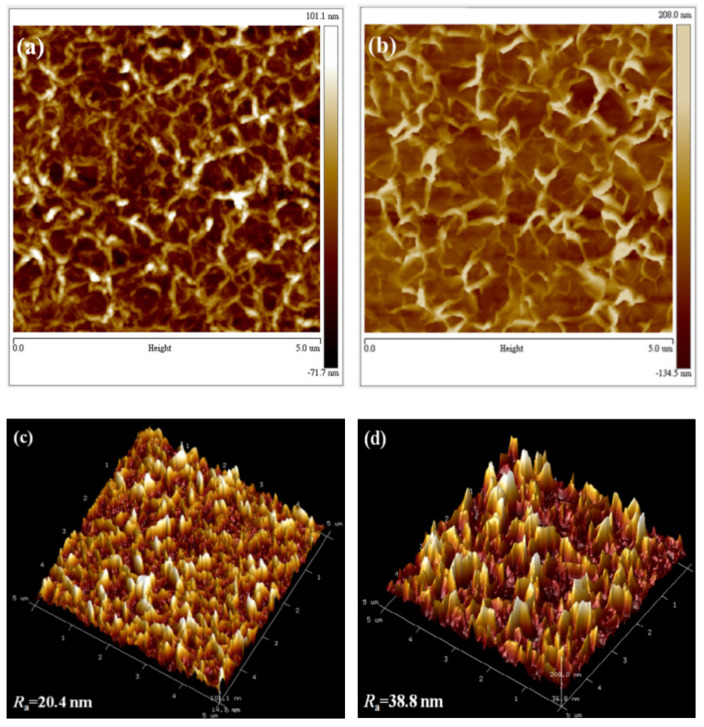
AFM topographic and 3D images of the Ni-doped ZnO NWLs with HMT concentrations of (**a***,***c**) 3 and (**b***,***d**) 5 mM.

To study the effects of the HMT concentration on the morphology of ZnO NWLs, the HMT molar concentration was varied from 1 to 5 mM, while the concentration of zinc nitrate hexahydrate was fixed at 25 mM. The top-view SEM images of the Ni-doped ZnO NWLs with 1, 3, and 5 mM HMT are depicted in Figure 2b and Figure 4a,b, respectively. All of these samples showed sponge-like nanostructures. It can be seen that the pores of ZnO NWLs significantly become larger as the HMT concentration increases. The cross-sectional SEM images of the Ni-doped ZnO NWLs with different HMT concentrations are displayed in Figure 2d and Figure 4c,d, respectively. As shown in Figure 4d, the ZnO NWLs with 5 mM HMT have the largest and intact nanosheets among three samples; smaller and denser nanosheets were formed when using 1 or 3 mM HMT. Based on these cross-sectional SEM images, the thickness of the Ni-doped ZnO NWLs with 1, 3 and 5 mM HMT is estimated to be 150, 175, and 250 nm, respectively. It was observed that ZnO nanosheets grew larger and intact as the HMT concentration increased. The reason to this phenomenon can be explained as follows. The hydrolysis of HMT in water produces OH^−^ hydroxyl ions which react with Zn^2+^ ions to form zinc hydroxide ions Zn(OH)_4_^2−^ [27]. Higher HMT concentration leads to more Zn(OH)_4_^2−^ ions in the solution to develop fast nucleation of ZnO crystallites. As the HMT concentration was increased to 7 mM, some flower-like aggregates formed (see Figure S1 in the supplementary information). Mirabella and coworkers proposed the growth kinetics and photocatalytic activity of ZnO NWLs [30], stating that a more interlaced structure and a higher density of NWLs were produced when incorporating extra ammonium hydroxide. Our study provides a different way to modify the morphology of ZnO NWLs by adjusting the HMT concentration. To the best of our knowledge, this is the first report that discusses relationship between the morphology of ZnO NWLs and HMT concentration. The AFM topographic and 3D images of the Ni-doped ZnO NWLs with HMT concentrations of 1, 3, and 5 mM are shown in Figure 3b,d and Figure 5a–d), respectively. It was found that AFM images of those ZnO NWLs are basically consistent with SEM observations. The R_a_ values of the Ni-doped ZnO NWLs with HMT concentrations of 1, 3, and 5 mM were 12.2, 20.4, and 38.8 nm, respectively. The surface roughness of ZnO NWLs was enlarged with increasing the HMT concentration, since the increased HMT concentration promoted fast nucleation of ZnO crystallites for the growth of NWLs. The EDS analysis confirms the existence of Zn, O, and Ni elements of the prepared Ni-doped ZnO NWLs using 1 mM HMT (see Figure S2 in the Supplementary Information), in addition to In and Sn from the ITO substrate and the Al deposited layer by thermal evaporation. The actual Ni doping content was 2.5 wt% in ZnO NWLs. The observation of Al signal in ZnO NWLs was also reported by Chu’s group when adopting the three-step deposition method [27].

### 3.2. Optical Measurements

**Figure 6:**
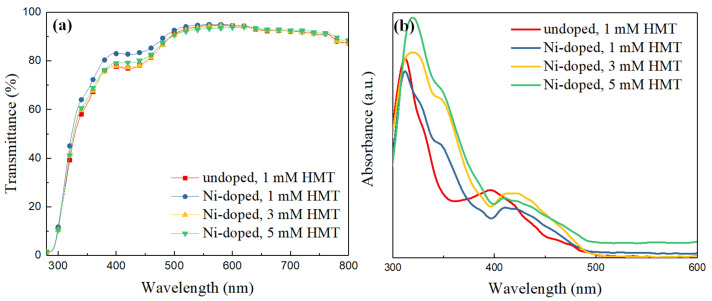
(**a**) Transmission and (**b**) absorption spectra of the undoped and Ni-doped ZnO NWLs with HMT concentrations of 1, 3, and 5 mM.

The transmission and absorption spectra of the undoped and Ni-doped ZnO NWLs from 300 to 800 nm are shown in Figure 6a,b, respectively. The transmittance of ZnO NWLs reaches 80% at 400 nm and nearly 90% in the range of 500–800 nm. The high transmittance of ZnO is beneficial for efficient photon input and/or output with reduced energy loss for different optoelectronic applications. The UV–Vis absorption spectra of different ZnO samples are depicted in Figure 6b. The absorption maximum of ZnO is located at 320 nm, while a shoulder at around 360 nm for those Ni-doped ZnO NWLs is observed, which is in agreement with the previous literature [12]. Furthermore, the absorbance of ZnO becomes stronger with increasing the HMT concentration because of more ZnO nanosheets formed in the hydrothermal bath [27].

### 3.3. Electrical Measurements

**Figure 7:**
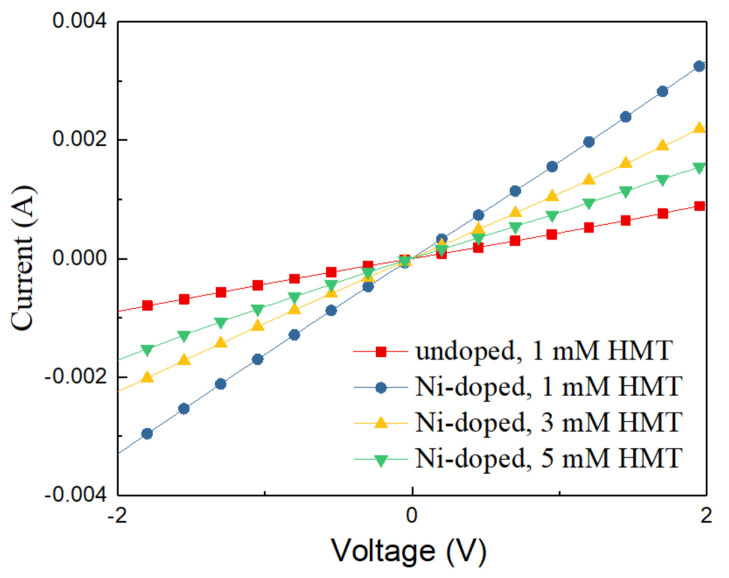
I–V characteristics of the electron-only devices based on the undoped and Ni-doped ZnO NWLs with HMT concentrations of 1, 3, and 5 mM.

To investigate the effect of Ni-doping and HMT concentration on the electrical conductivity of ZnO NWLs, electron-only devices with the structure of ITO/undoped or Ni-doped ZnO NWLs/Au were fabricated, and the corresponding I–V characteristics are shown in Figure 7. It is clearly seen that the devices based on the Ni-doped ZnO NWLs possess greater slope and higher electrical conductivity than that of the undoped one, indicative of enhanced electron transport ability for the Ni-doped ZnO NWLs. Moreover, the electrical conductivity of the Ni-doped ZnO NWLs with the HMT concentration of 1 mM was higher than with 3 or 5 mM HMT. This is because higher HMT concentration facilitates faster nucleation of ZnO that leads to an increment in the total thickness. With regard to the future application of the synthesized ZnO nanomaterials, both the amounts of Ni dopant and HMT should be carefully controlled to achieve high device performance.

### 3.4. Electrochemical Measurements

**Figure 8:**
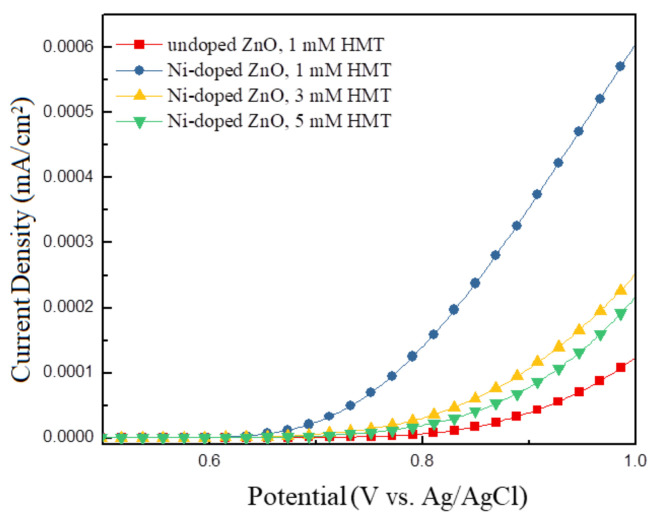
LSV curves for the undoped and Ni-doped ZnO NWLs with HTM concentrations of 1, 3, and 5 mM.

To evaluate the electrochemical activity of the undoped and Ni-doped ZnO NWLs, I–V characteristics of the four samples in a three-electrode cell were measured. In this part, we adopted LSV experiments to evaluate the activity of the catalyst for hydrogen and/or oxygen evolution reactions. Some bubbles were produced near the working electrode of the prepared ZnO NWLs when the bias voltage was applied. The LSV was measured to investigate the correlation between current densities and bias voltage, as shown in Figure 8. It can be seen that the undoped ZnO NWLs exhibited lower current density than the other three Ni-doped ZnO NWLs. The prepared Ni-doped ZnO NWLs exhibited better electrochemical activity, which could be attributed to Ni doping to enhance electron transport ability of ZnO. Particularly, the Ni-doped ZnO NWLs with the HMT concentration of 1 mM revealed 6-fold higher current density at 1 V bias compared with the undoped ZnO NWLs, which demonstrates the best electrochemical performance due to the largest surface area and enhanced electron transport ability. It is reported that nanostructured catalyst has more active sites per geometric area to facilitate diffusions of ions, electrolytes, and generated gas [34]. Apart from our ZnO NWLs, graphdiyne NWLs have also been utilized as the catalyst for water splitting [35]. The structure-controlled graphdiyne NWLs showed effective application in water oxidation, revealing a current density of 0.25 mA/cm^2^ at a bias of 1 V and realizing significantly improved electrochemical activity and stability. Liu et al. utilized ZnO nanorods array as the catalyst for LSV measurement, receving a current density of 0.2 mA/cm^2^ by applying a bias of 1 V [36]. Compared with those reports, the prepared Ni-doped ZnO NWs in this study possessed a much lower current response that should be further optimized. It is concluded that the design and synthesis of porous nanomaterials is a key issue to achieve efficient electrochemical performance with enlarged surface area and electrical conductivity.

## 4. Conclusions

The 3 mol% Ni-doped ZnO NWLs with sponge-like nanostructures on the ITO substrates were successfully prepared via the modified hydrothermal method through changing the HMT concentration from 1 to 5 mM. The incorporation of Ni atoms into ZnO NWLs lattices was verified by UV-Vis absorption and EDS experiments. The pores of Ni-doped ZnO NWLs are obviously smaller than those of the undoped one. Furthermore, Ni-doping helps to decrease surface roughness of ZnO NWLs. Besides, the pore size of ZnO NWLs can be adjusted by changing the HMT concentration, i.e., the pores become larger as the HMT concentration increases. However, the surface roughness of ZnO NWLs was enlarged when increasing the HMT concentration due to fast nucleation of ZnO crystallites. Through I–V measurement of electron-only devices, it can be found that Ni-doping and HMT incorporation show opposite effects on the electrical conductivity of devices. Owing to higher electron transport ability of the Ni-doped ZnO NWLs, an enhancement in electrochemical behavior has been demonstrated through water splitting.

## Supplementary Materials

The following are available online at https://www.mdpi.com/article/10.3390/nano11081980/s1, Figure S1: Top-view SEM image of the Ni-doped ZnO NWLs with an HMT concentration of 7 mM, Figure S2: EDS result showing the element composition of Ni-doped ZnO NWLs using 1 mM HMT.
