# Supplementary material for: Nickel-Doped ZnO Nanowalls with Enhanced Electron Transport Ability for Electrochemical Water Splitting
Source: Nanomaterials (Basel). 2021 Jul 31;11(8):1980. doi: 10.3390/nano11081980 (PMC8398548; doi:10.3390/nano11081980)
Supplement: Supplementary file 1 [file nanomaterials-11-01980-s001.zip › nanomaterials-1313432-supplementary.pdf]

Supplementary Information

Nickel-Doped ZnO Nanowalls with Enhanced Electron Transport Ability for Electrochemical Water Splitting

Bing-Chang Jiang and Sheng-Hsiung Yang *

Institute of Lighting and Energy Photonics, College of Photonics, National Yang Ming Chiao Tung University,
city postcode, country; ben0658228@gmail.com
*   Correspondence: yangsh@mail.nctu.edu.tw

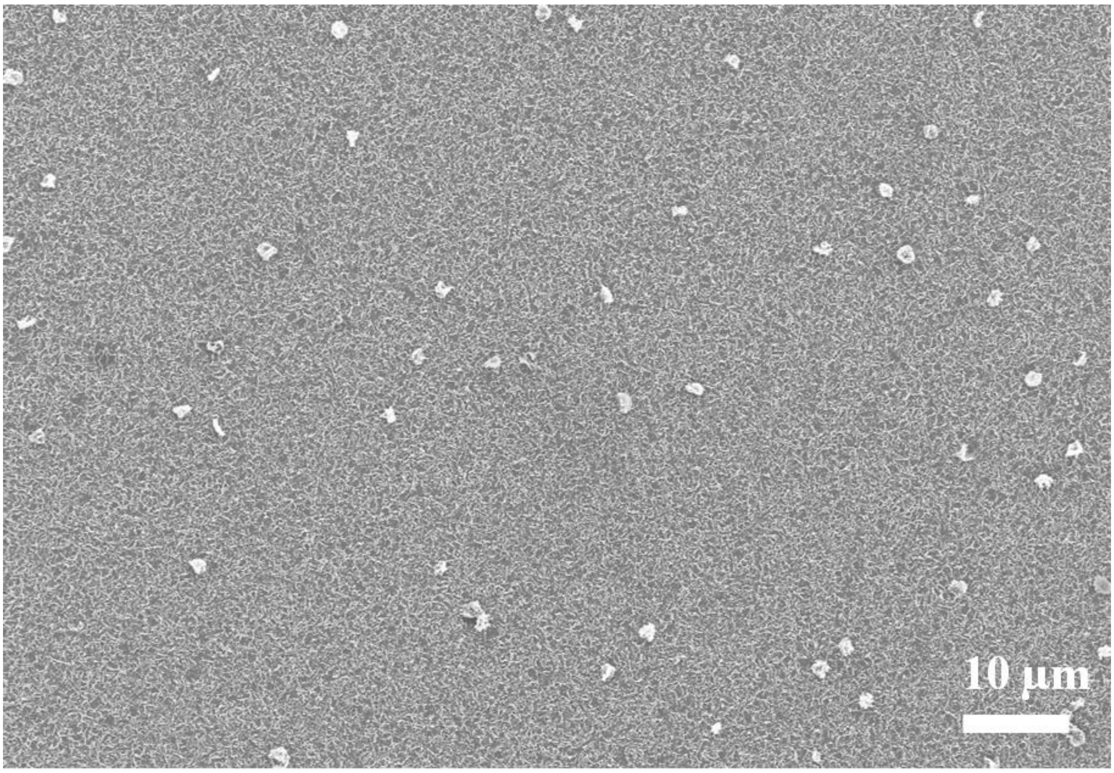

Figure S1. Top-view SEM image of the Ni-doped ZnO NWLs with HMT concentration of 7 mM.

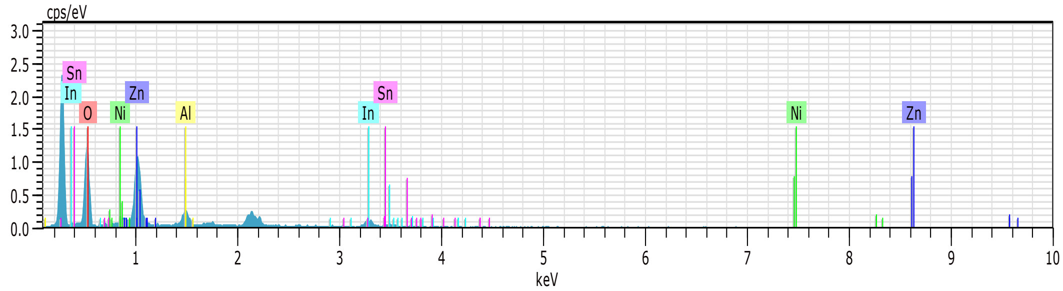

Figure S2. EDS result showing the element composition of Ni-doped ZnO NWLs using 1mM HMT.
